# Efficacy of live feedback to improve objectively monitored compliance to prescribed, home-based, exercise therapy-dosage in 15 to 19 year old adolescents with patellofemoral pain- a study protocol of a randomized controlled superiority trial (The XRCISE-AS-INSTRUcted-1 trial)

**DOI:** 10.1186/s12891-016-1103-y

**Published:** 2016-06-02

**Authors:** Henrik Riel, Mark Matthews, Bill Vicenzino, Thomas Bandholm, Kristian Thorborg, Michael Skovdal Rathleff

**Affiliations:** Center for Sensory-Motor Interaction (SMI), Department of Health Science and Technology, Faculty of Medicine, Aalborg University, Fredrik Bajers Vej 7D, 9220 Aalborg East, Denmark; Research Unit for General Practice in Aalborg and Department of Clinical Medicine, Aalborg University, Fyrkildevej 7, 9220 Aalborg East, Denmark; School of Health and Rehabilitation Sciences: Physiotherapy: Sports Injury Rehabilitation and Prevention for Health, The University of Queensland, St. Lucia, QLD 4072 Australia; Physical Medicine & Rehabilitation Research – Copenhagen (PMR-C), Department of Physical Therapy, Department of Orthopedic Surgery, Clinical Research Centre, Copenhagen University Hospital, Amager-Hvidovre, Copenhagen, Denmark; Sports Orthopedic Research Center - Copenhagen (SORC-C). Department of Orthopedic Surgery, Copenhagen University Hospital, Amager-Hvidovre, Copenhagen, Denmark; Department of occupational therapy and physiotherapy, Aalborg University Hospital, Hobrovej 18-22, 9100 Aalborg, Denmark

**Keywords:** Patellofemoral pain, Adolescents, Exercise, Feedback, Time under tension, Compliance

## Abstract

**Background:**

Patellofemoral pain is one of the most frequent knee conditions among adolescents with a prevalence of 7 %. Evidence-based treatment consists of patient education combined with hip and quadriceps strengthening. Recent evidence suggests that a large proportion of adolescents does not follow their exercise prescription, performing too few repetitions or too fast below the prescribed time under tension. Live feedback, such as a metronome or exercise games, has previously shown promising results in improving the quality of exercises. The aim of this study is to investigate if live feedback from a sensor (BandCizer™) and an iPad will improve the ability of adolescents with PFP to perform exercises as prescribed.

**Methods:**

This study is a randomized, controlled, participant-blinded, superiority trial with a 2-group parallel design. Forty 15 to 19 year old adolescents with patellofemoral pain will be randomized to receive either live visual and auditory feedback on time under tension or no feedback on time under tension during a 6-week intervention period. Adolescents will be instructed to perform three elastic band exercises. Feedback will be provided by BandCizer™ and an iPad. The adolescents perform the exercises twice a week unsupervised and once a week during a supervised group training session. The primary outcome will be the mean deviation of the prescribed time under tension per repetition in seconds during the course of the intervention.

**Discussion:**

Low compliance is a major problem among adolescents with patellofemoral pain. Providing the adolescents with real time feedback on time under tension from a sensor and an iPad could potentially help the adolescents perform the exercises as prescribed. This may increase the total exercise dosage they receive during treatment which may help improve patient outcomes.

**Trial registration:**

Registered at ClinicalTrials.gov (identifier: NCT02674841) on February 4^th^ 2016.

## Background

Thirty percent of adolescents between 15 and 19 years old report having knee pain [1]. Patellofemoral Pain (PFP) is one of the most frequent knee conditions among adolescents and has a prevalence of 7 % [2, 3]. Patients usually describe their pain as being diffuse anterior knee pain that is aggravated by sitting for prolonged periods, climbing or descending stairs, running or squatting [2].

Evidence-based treatment of PFP consists of patient education combined with hip and quadriceps strengthening [2, 4]. A meta-analysis based on studies of PFP in adults reported a positive effect of multimodal treatment with 62-84 % being fully recovered 12 months after the treatment [5]. The latest review on PFP in adolescence and adulthood indicates that treatment seems to have a somewhat lower effect in adolescence [6]. The reason for this is unknown, but compliance seems to play an important role [7].

Hip and quadriceps exercises have a better effect if the exercises are performed more frequently [7, 8], however a large proportion of adolescents do not follow their exercise prescription [7] or they perform the exercise too fast with too few repetitions thus not performing the prescribed exercise dose (Rathleff et al. *in review*). Exercise parameters such as load, time under tension (TUT), range of motion (ROM), the number of repetitions and sets being performed collectively influence the total exercise dosage patients receive during rehabilitation [9]. The question is if live feedback during exercises may improve compliance by helping adolescents perform the exercise as prescribed and, thus, helps them achieve the prescribed exercise dosage.

Previous research has shown promising results in various populations when using a metronome for guidance [10, 11] or exercise games as live feedback [12] and thereby improving the quality of exercises. Some shortfalls of these strategies are that a metronome does not give feedback on the exercises performed and exercise games often require a lot of space in order to be able to capture the user performing the exercises by camera.

One way to provide adolescents with live feedback on the quality of home-based elastic band exercises is by using a sensor called BandCizer™ (BandCizer Aps, Denmark). A systematic review on self-reported compliance by Bollen et al. identified a need for a valid instrument that can measure compliance to prescribed home-based exercises [13]. Pertaining to this need, BandCizer™ is a valid tool that can quantify compliance and measure the number of repetitions performed, TUT and the force used to stretch the elastic band (pulling force). BandCizer™ consists of two parts that are mounted on either side and held together by internal magnets. It transmits data to an iPad with the BandCizer™-app [14, 15]. The app can supply the user with live feedback on TUT and pulling force. This exercise-integrated system may thus help the adolescents with PFP to perform the exercises as instructed.

### Purpose

The purpose of this study is to investigate if live feedback on TUT during home-based exercises will improve the ability to perform the exercises with the prescribed TUT per repetition compared with no feedback on TUT among adolescents with PFP during a 6-week intervention.

### Hypothesis

Hypothesis: adolescents who receive live feedback on TUT from BandCizer™ (feedback group) will have a significantly lower mean deviation from the prescribed TUT compared to the group not receiving feedback on TUT (controls) during the course of the intervention.

## Methods

### Study design

This study, which is called the The XRCISE-AS-INSTRUcted-1 trial, is a randomized, controlled, participant-blinded, superiority trial, with a 2-group parallel design to be conducted in Aalborg, Denmark. The primary endpoint will be 6 weeks after an initial exercise instruction, using a summary of exercise dosage-data collected from 0 to 6 weeks. Reporting of this study will follow CONSORT guidelines for reporting parallel group randomized trials with the extension for non-pharmacological treatments and TIDieR for intervention description [16, 17]. Reporting of this protocol will follow the SPIRIT statement [18]. The study will be conducted at the Department of Occupational Therapy and Physiotherapy at Aalborg University Hospital in Denmark.

### Recruitment

Forty 15 to 19 year old adolescents with PFP will be recruited from local GP clinics. They will be contacted by telephone where they will be invited to participate in an interview regarding their knee pain. The telephone screening process contains questions about the duration and history of their knee pain. Those adolescents whose history indicates PFP will be invited to attend a clinical examination together with their legal guardian at Aalborg University Hospital. The clinical examination and decision to include the participants will by made by HR. If the recruitment from the GP clinics is insufficient, the study will be advertised on social media as well as students from 4 upper secondary schools in Aalborg will be invited to answer an online questionnaire regarding self-reported knee pain. Adolescents who report knee pain will then be contacted by telephone and will participate in the same telephone screening process as the adolescents who are recruited from the GP clinics.

### Eligibility criteria

Eligibility criteria are in line with a previous study of this age group [7]:

Inclusion criteria:

- 15 to 19 years of age
- Anterior knee pain of non-traumatic origin which is provoked by at least two of the following activities: prolonged sitting with bent knees or kneeling, squatting, running, jumping or ascending or descending stairs
- Tenderness on palpation of the peripatellar borders
- Pain of more than 6 weeks’ duration
- Self-reported worst pain during the previous week ≥ 30 mm on a 100 mm Visual Analog Scale (VAS)

Exclusion criteria:

- Concomitant pain from other structures in the knee (e.g. ligament, tendon or cartilage), the hip or the lumbar spine
- Previous knee surgery
- Patellofemoral joint instability

A registered physiotherapist (HR) with four years of clinical experience in treating patients with musculoskeletal conditions will be in charge of selecting participants and instructing them during the supervised training sessions.

### Intervention

**Fig. 1:**
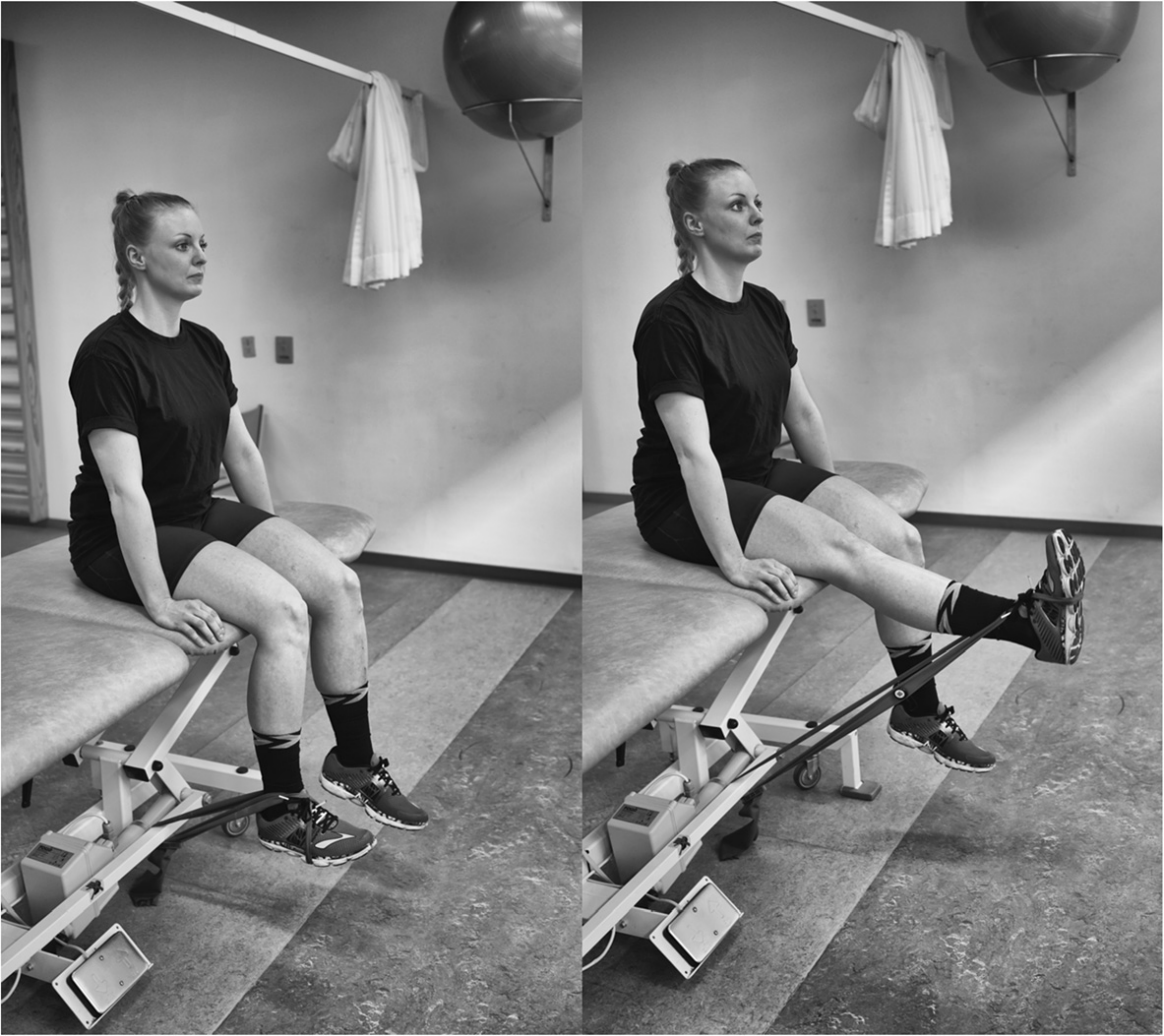
Knee extension. The subject provided consent to appear

**Fig. 2:**
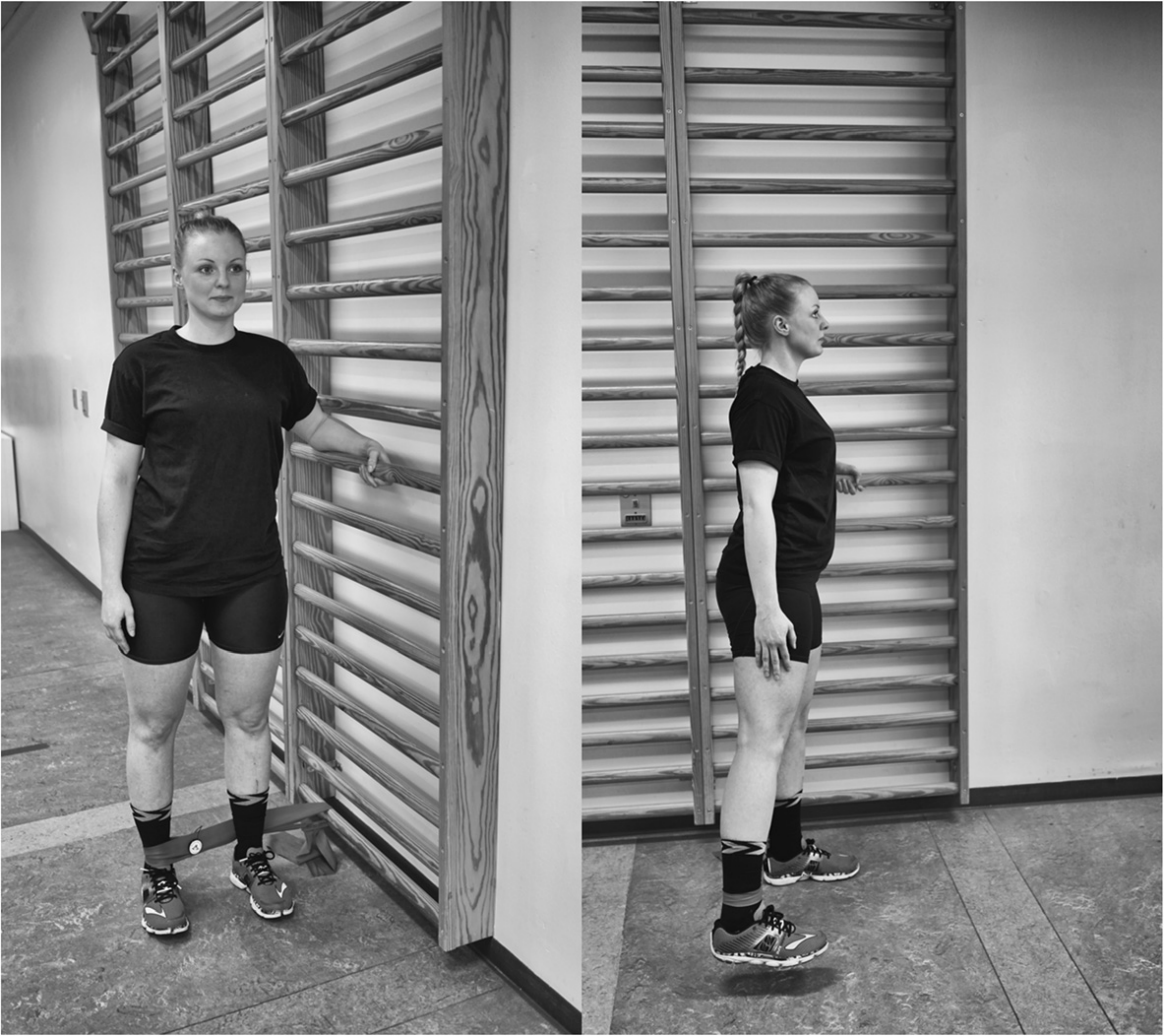
Hip abduction. The subject provided consent to appear

**Fig. 3:**
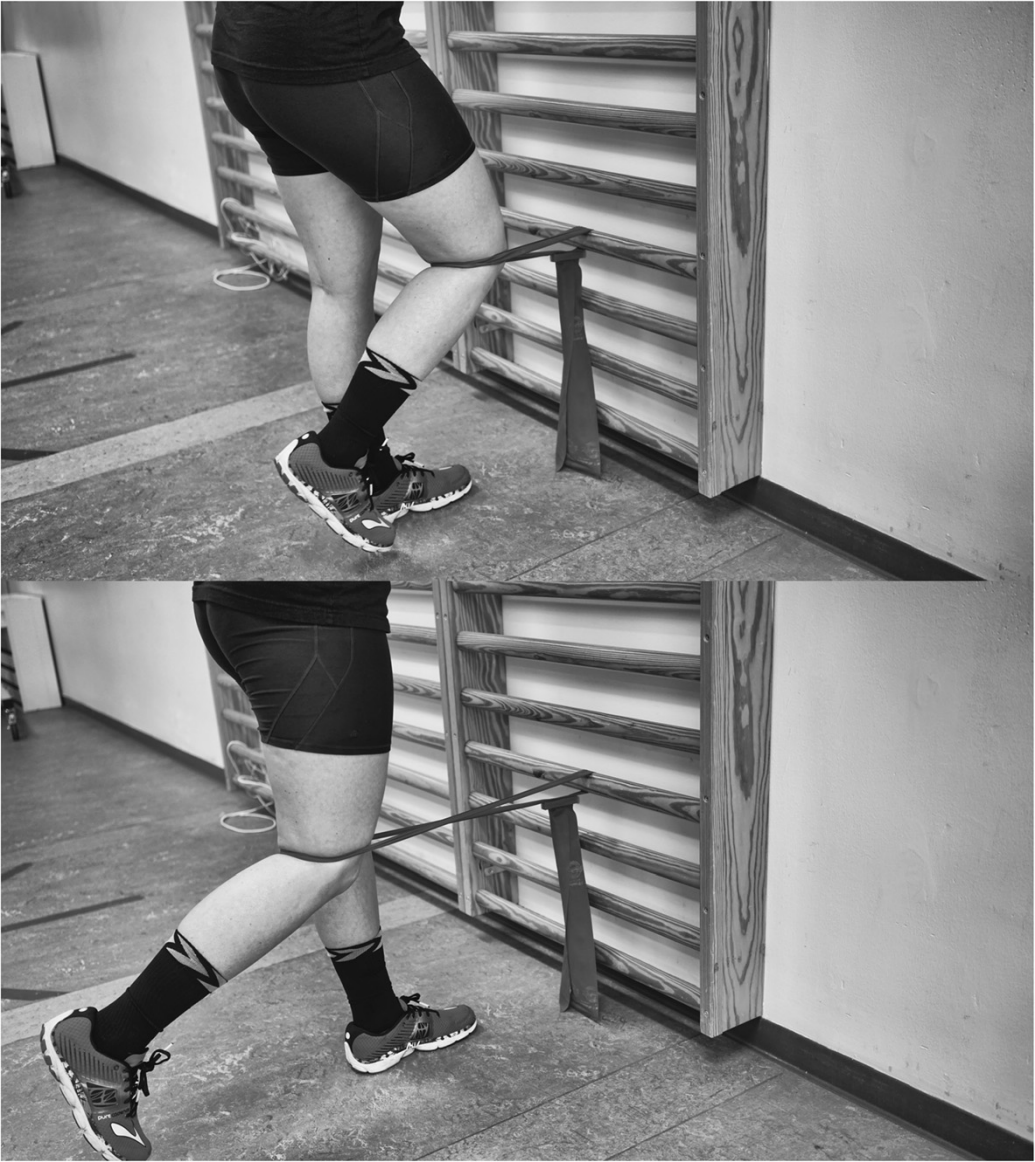
Hip extension. The subject provided consent to appear

Once informed consent has been gained, the participants will be instructed in performing three exercises with an elastic band; knee extension (Fig. 1), hip abduction (Fig. 2) and hip extension (Fig. 3). These types of exercises have previously been tested and found effective in patients with PFP [2, 19, 20]. The exercise descriptors, which are adopted from the mechano-biological descriptors from Toigo and Boutellier [21], are described in the table below (Table 1).

**Table 1:** Exercise descriptors

	Knee extension (Fig. 1)	Hip abduction (Fig. 2)	Hip extension (Fig. 3)
1. Load magnitude	10-12 RM	10-12 RM	10-12 RM
2. Number of repetitions	10	10	10
3. Number of sets	3	3	3
4. Rest in between sets	1 min 50 seconds	1 min 50 seconds	1 min 50 seconds
5. Number of exercise interventions (per (day) or week)	3/week	3/week	3/week
6. Duration of the experimental period ((day) or weeks)	6 weeks	6 weeks	6 weeks
7. Fractional and temporal distribution of the contraction modes per repetition and duration (s) of one repetition	3 s concentric	3 s concentric	3 s concentric
	2 s isometric	2 s isometric	2 s isometric
	3 s eccentric	3 s eccentric	3 s eccentric
8. Rest in-between repetitions ((s) or (min))	No	No	No
9. TUT ((s) or (min))	8 s/repetition	8 s/repetition	8 s/repetition
	80s/set	80s/set	80s/set
	4320 s/total intervention	4320 s/total intervention	4320 s/total intervention
10. Volitional muscular failure	Yes	Yes	Yes
11. Range of motion	90° to 180^o^	0° to 45^o^	45° hip flexion to 0° hip flexion/extension
12. Recovery time in-between exercise sessions ((h) or (d))	48 h	48 h	48 h
13. Anatomical definition of the exercise (exercise form)	Knee extension is performed with the participant sitting in 90^0^ knee flexion. The elastic band is looped around the ankle and around a solid anchor near the floor under the participant (Fig. 1). The knee is extended to approximately 180^0^.	Hip abduction will be performed in standing with the elastic band looped around the ankle and anchored to the wall at ankle height. The stance leg will be in front of the band and the target hip in a slight internal rotation (Fig. 2). Hip abduction will then be performed to approximately 45^0^.	Hip extension will be performed in standing with target hip in 45^0^ hip flexion. One end of the elastic band fixated at knee height and looped around the back of the knee (Fig. 3). The hip is then extended to approximately 0° hip extension whilst maintaining a neutral lumbo-pelvic position.

Participants will receive an elastic band, a BandCizer™ and an iPad with the BandCizer™-app. Before and after each exercise they are instructed to record their knee pain on a 100 mm VAS that is integrated in the app. They are instructed to perform the exercises three times each week during the 6-week intervention. Twice a week the exercises are performed at home whilst the last exercise session will be a group training session supervised by a physiotherapist. In connection with the initial instruction of the exercises, 10-12 RM will be determined by shortening the elastic band to a length where the participants feel that they will not be able to perform more than 10 repetitions. The pulling force exerted when the exercise is performed correctly will be measured by the BandCizer™ and recorded by the investigator. If the participants become able to perform more than 10 repetitions during the intervention period, they will be instructed to shorten the band or change to a different grade of band. In this situation a new measurement of recommended pulling force will be made.

The feedback group will have access to live visual and auditory feedback on TUT and pulling force from the BandCizer™-app when they perform the exercises. Controls will only receive visual feedback on pulling force.

### Withdrawal and adverse events

If a participant experiences an adverse event (e.g. an injury to the musculoskeletal system such as a muscle tear, a muscle strain, a sprained joint, injury from falling, DOMS that lasts for more than 48 h after performing the exercises or exacerbation of PFP) and is not able to perform the exercises, the participant will be able to withdraw from the study. The study may also be discontinued by participant request or withdrawal of informed consent. Data until the point of withdrawal will be included in the data analyses. If a participant experiences an adverse event and has to withdraw, data until the last training before the adverse event occurred will be included in the analyses. The participants are instructed to report any adverse events to the primary investigator as quickly as possible either by e-mail, SMS, phone call or during the supervised training sessions. The primary investigator will then ask the participant if the event occurred when they performed the exercises or during other activities. If the event occurred during the exercises the primary investigator will report the incident to the sponsor as quickly as possible and no later than 15 days after the participant reported the event. Sponsor will report adverse events to the Ethics Committee of North Denmark Region no later than 7 days after being informed.

### Compliance and participant retention

Compliance refers to whether or not an individual conforms to the recommendations of the prescribed dosage, timing and frequency of an intervention [22].

Participants of both groups will be told initially and during group training sessions throughout the study that compliance to exercises are important and will improve their odds of recovery. They will be told that compliance consists of performing the prescribed number of repetitions, the pulling force and the TUT.

**Fig. 4:**
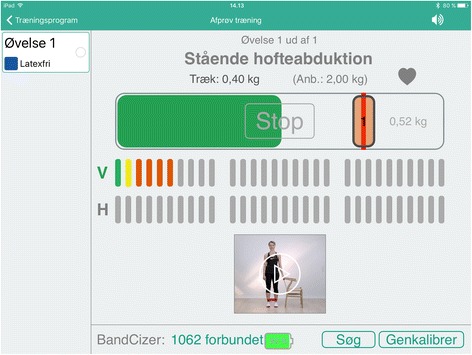
BandCizer-app with feedback on TUT

**Fig. 5:**
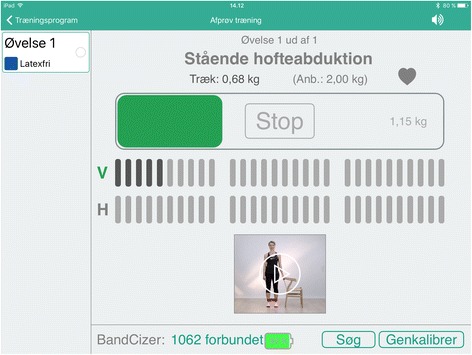
BandCizer-app without feedback on TUT

Using BandCizer™ could improve compliance as the participants know that their performance is being recorded. The feedback group receives visual feedback and auditory guidance from the iPad when performing the exercises and have access to a calendar with the planned training sessions. The visual feedback consists of a vertical bar that moves from side to side on a horizontal bar at the same pace as the predetermined TUT. When the elastic band is stretched the horizontal bar is being filled with colour and the participant has to keep up with the vertical bar to perform the exercise with the prescribed TUT and pulling force (Fig. 4). The auditory guidance consists of a voice that counts the seconds of each contraction phase. The control group has access to the calendar as well, however they only receive visual and auditory feedback on pulling force and has no vertical bar to keep up with during the repetitions (Fig. 5). Both groups are asked to record pain on a 100 mm VAS scale before and after each exercise.

All group training sessions will be planned before the intervention starts for all participants. If they are unable to attend any of the sessions, they are asked to contact the primary investigator by phone or e-mail and they will then be instructed to do the exercises at home instead. If any participant fails to show up for a training session and did not cancel beforehand they will be contacted by the primary investigator and asked in a friendly manner if they will return to the next group training session and they will be asked to do the missing training session at home.

### Information and patient education

Participants will be instructed not to do any type of other strengthening exercises for the lower extremities during the intervention and not to consult any physicians or physiotherapists because of their knee pain. They will be advised to continue participating in physical activity as long as: (a) their pain is no higher than 30 mm on a 100 mm VAS during the activity, (b) their knee pain does not outlast the physical activity, and (c) there is no strong increase in symptoms post activity. Participants will be told to register any use of analgesic or anti-inflammatory substances.

### Outcomes

As we are most interested in the exercise dose performed by the participant our primary outcome will involve TUT. We will also collect a range of secondary outcomes pertaining to the exercise dose, such as, number of repetitions, pulling force achieved during each repetition, and muscle strength. The exercises being performed have been shown to change condition status and so we will collect measures of pain, disability and a global rating of change, but these are secondary to our primary goal of evaluating the effects of feedback on TUT. The schedule for assessments is found in the SPIRIT figure (Table 2).

**Table 2:**
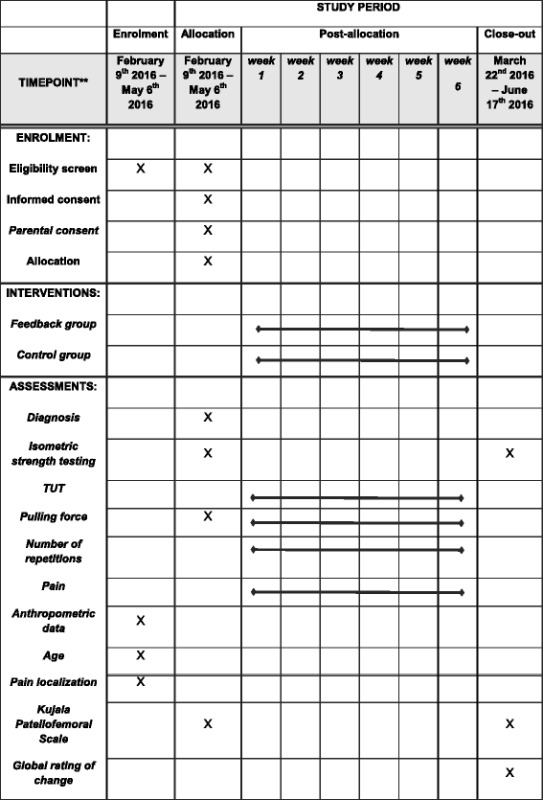
SPIRIT figure. Schedule of enrolment, interventions and assessments

Primary outcome:

- Mean deviation from the prescribed TUT per repetition in seconds during the course of the intervention. The mean deviation is calculated as the difference between actual TUT and prescribed TUT per repetition (8 s). E.g. if the actual TUT is 6.5 s, the deviation is 1.5 s for this repetition. This is chosen as the primary outcome as TUT plays a large role in the total training dosage [21, 23–25] and adolescents have previously shown difficulties performing exercises with the prescribed TUT (Rathleff et al. *in review*).

Secondary outcomes:

- The total number of repetitions performed during the intervention period.
- Pulling force exerted per repetition measured in kilos. This will be expressed as the maximum pulling force during each repetition and corresponds to the resistance used when calculating training volume [26].
- Isometric strength (presented as Nm/kg) of knee extension, hip extension and hip abduction. Isometric strength will be collected at baseline and follow-up and will be used to explore the association between total exercise dose and changes in isometric strength. Isometric strength will be recorded at baseline and post intervention using a dynamometer (Commander PowerTrack, JTECH Medical, Midvale, Utah, USA) and will follow the protocol by Rathleff et al. [27] for knee extension (ICC= > 0.92). The test for hip abduction and hip extension will follow the test positions of the side-lying hip abduction (ICC = 0.76) and the hip extension with a short lever (ICC = 0.81) described by Thorborg et al. [28].
- Pain measured on a 100 mm VAS, where 0 mm is no pain and 100 mm is worst pain imaginable. The participants will enter their rating of pain intensity into the BandCizer™-app before and after each exercise. Pain is chosen as an outcome measure to investigate if the participants are more likely to comply to exercises that cause less pain.
- Kujala Patellofemoral Scale-score (0-100 score, with 0 as complete disability and 100 as fully functional). The Kujala Patellofemoral Scale is a frequently used validated outcome measure in PFP [19, 29, 30] however, it does not come in a Danish version. Therefore a Danish translation has been made with the same content and wordings as the original English version and a backwards translation to English was made according to principles of the translation of patient-reported outcome measures [31], however the translation has not been published. Kujala will be collected at baseline and at follow-up.
- Global rating of change at follow-up. This will be used to measure the participants’ self-reported recovery on a 7-point Likert scale ranging from “much improved” to “much worse”. Participants are categorised as improved if they rate themselves as “much improved” or “improved” (category 6-7) and categorised as not improved if they rate themselves from “slightly improved” to “much worse” (category 1-5). A 7-point Likert scale to assess self-reported change from baseline has previously been used in studies resembling this study [7, 19].

### Sample size and power considerations

It is expected that the feedback group will have a TUT close to the recommended 8 s per repetition whilst the control group will have a TUT of 6.5 s which is close to the results of an unpublished study (Rathleff et al. *in review*). Based on a standard deviation of 1.22, which was found in the before-mentioned study, and a two-sided 5 % significance level and a power of 80 %, a sample size of 15 participants per group will be necessary. Taking into consideration possible drop-outs, we will include a total of 40 participants.

### Randomisation

Forty adolescents diagnosed with PFP will be block randomized in block sizes of 2 to 8 (1:1) into 2 parallel groups of 20 participants using a random number generator on www.random.org. A researcher not involved in the study will generate the allocation sequence and is the only person who will know the block sizes. In practice, after all baseline measurements have been made, the primary investigator will take a sequentially numbered opaque sealed envelope in which allocation to either the feedback group or the control group has been previously determined.

### Blinding

Participants will be told that the study is about adolescents with PFP and training with a new sensor, BandCizer™, that can give information about how they train, and that there will be two groups that use the BandCizer™-app in two different ways. They will not receive any information about the primary outcome measure or how the parallel group uses the app and will thus be blinded to the type of exercise feedback. The two groups will be training in separate group training sessions.

### Data collection and management

**Fig. 6:**
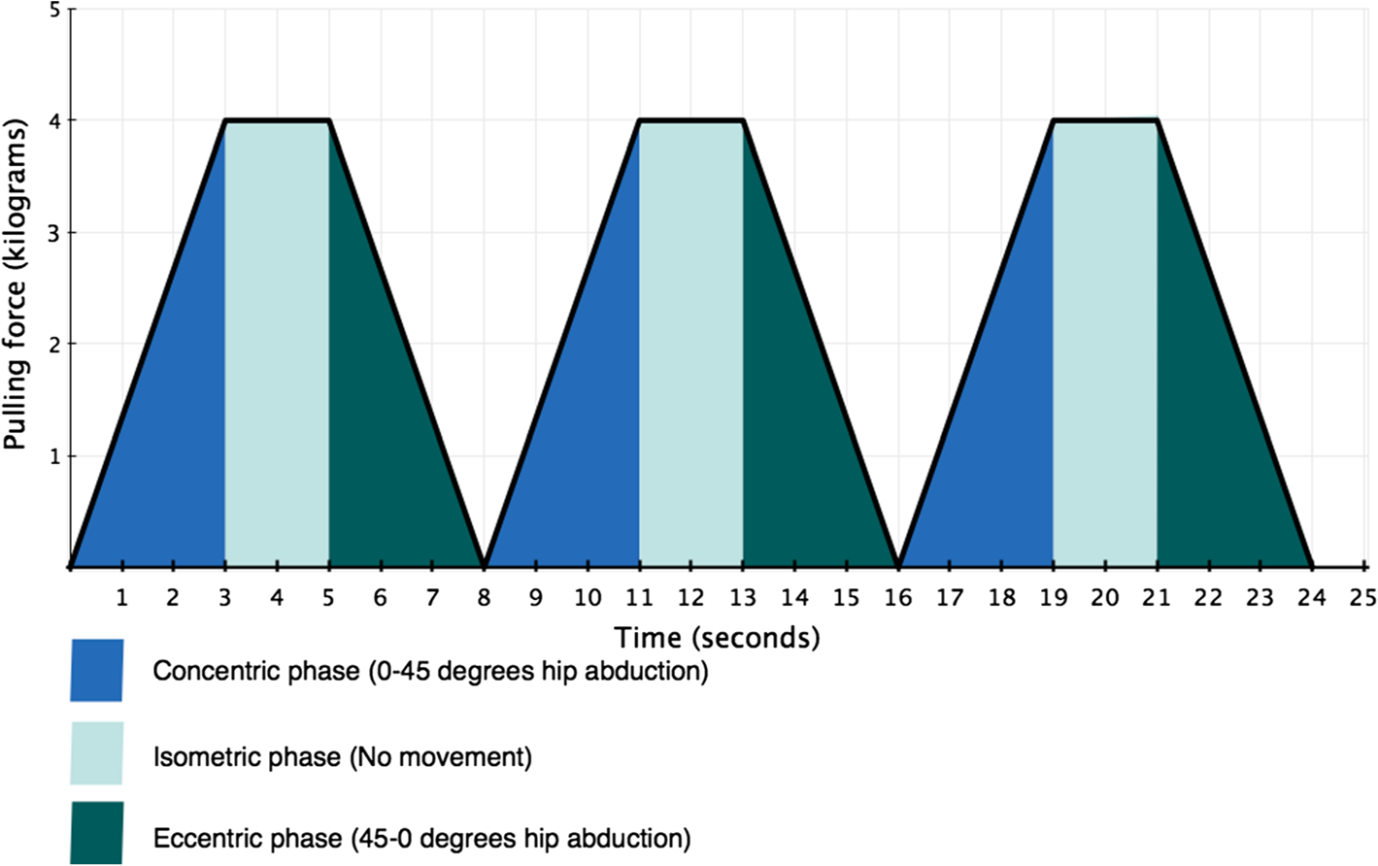
Visualisation of three consecutive repetitions of hip abduction with the prescribed TUT

All data will initially be written on paper forms and afterwards entered into Microsoft Excel 2013 (Microsoft Corporation, Washington, USA) by the primary investigator at the study site where data originated. Data from BandCizer™ is uploaded from the iPad to an online server from where raw data on TUT, pulling force, repetitions and pain will be accessible only for the primary investigator. A visualisation of the data output from BandCizer™ is seen in Fig. 6. Prior to the statistical analyses data will be exported to IBM SPSS Statistics ver. 23 (IBM, New York, USA). Participants will complete all self-report data forms. All original paper forms and informed consent forms will be kept in a locked cabinet at the study site. All data will be kept for 10 years after completion of the study which in accordance with The European Code of Conduct for Research Integrity [32].

### Statistical analysis

All statistical analyses will be performed according to a pre-established analysis plan. IBM SPSS Statistics ver. 23 will be used as statistical software. The primary analysis will test the between-group difference of the mean deviation from the prescribed TUT per repetition using an unpaired *t*-test. Secondary analyses will test total repetitions, total pulling force per exercise, isometric strength and Kujala Patellofemoral Scale using an unpaired *t*-test. The relative risk (RR) will be calculated for the dichotomized global rating of change to determine the probability of being improved and a Z-test will test the proportion of repetitions that are performed with the prescribed TUT compared with the proportion of repetitions that deviate from the prescribed TUT. Explorative analyses using Pearson’s correlation coefficient will be performed to test: (a) the association between pain and compliance to a specific exercise, and (b) the association between total exercise dose and isometric strength progression.

### Analysis population and missing data

Participant data will be analysed on an intention-to-treat (ITT) basis as per group allocation. Any missing repetitions from the prescribed number of repetitions will be interpreted as non-compliance. Mean values of data until the point of drop out will be imputed for the remaining intervention for participants who drop out. If the participant has not made a single repetition, the group mean value will be imputed for this participant.

### Monitoring

A data monitoring committee will not be established as the intervention uses exercises that are commonly used in the population of interest and do not pose a threat to the participants. This exercise intervention has previously been used for adolescents with this type of knee condition and no adverse effects as a result of the exercise intervention have been reported. It involves exercises that are under the volitional control of the participant. They tolerate it well and there will be no stopping rules planned.

### Access to the final trial data set

The primary investigator and all co-authors will have unlimited access to the final data set before publication. The data, containing the de-identified individual patient data, will be publically available no later than 6 months after publication, consistent with the recent proposal by the International Committee of Medical Journal Editors (ICMJE) [33].

## Discussion

Patellofemoral pain is one of the most frequent knee conditions among adolescents and has a prevalence of 7 % [2, 3]. Treatment seems to be less efficacious in adolescence compared to adulthood [6] and compliance has been shown to play a role, probably because a large proportion of adolescents do not follow their exercise prescription [7]. The XRCISE-AS-INSTRUcted-1 trial will add knowledge as to whether or not visual and auditory feedback on TUT will improve the ability to perform exercises as prescribed.

The specific exercises have been chosen based on a recommendation of strengthening the hip and quadriceps muscles [2, 4]. In addition, activation of the trunk muscles has also been found beneficial in treating PFP [34, 35] and therefore free-standing hip exercises have been chosen to increase trunk activation compared to non-standing hip exercises such as the side-lying hip abduction. In order to be able to measure compliance with the BandCizer™ the participants need to use elastic bands as resistance instead of dumbbells or training machines however, elastic bands provide similar muscle activation as dumbbells [36].

### Strengths

The recruitment and study design has several strengths. Firstly, the use of BandCizer™ provides an objective measure of compliance which was requested in the latest systematic review on self-reported compliance to home-based exercise programs [13]. A recent study showed that adolescents reported a 2.3 times higher exercise dosage in their exercise diary compared to TUT data from the BandCizer™ (Rathleff et al. *in review*)*.* This highlights the importance of an objective measure of compliance. Secondly, the participant blinding of primary outcome measures will ensure that the participants do not focus more on performing the exercises as prescribed than they would when receiving standard exercise prescription by a physiotherapist. Thirdly, the inclusion criteria used in this study are in line with those of previous studies of this patient group [3, 7, 29, 34] which will make comparisons across studies easier. Fourthly, reporting the study protocol using the SPIRIT statement, and outlining the specific exercise descriptors, which has been requested in a recent review on exercise interventions for patients with chronic conditions by Hoffmann et al. [37], facilitates the replication of the study’s findings and its translation into clinical practice. Fifthly, the intervention resembles current physiotherapy practice by combining home-based and supervised training sessions, which increases the external validity.

### Limitations

The participants know that they participate in a study and their exercises are somehow being recorded. This may lead to an increased compliance that does not represent normal clinical practice, however, this limitation will apply to both the feedback group and the controls. The previous study on BandCizer™ and adolescents with PFP by Rathleff et al. (Rathleff et al. *In review*) found a low compliance in spite of the fact that the participants received feedback on pulling force. This emphasises that compliance may not be influenced by using BandCizer™ and receiving feedback on pulling force alone. Another theoretical limitation is that BandCizer™ can only record what is occurring at the exercise band and not who is performing the exercises or if they are being performed with other body parts.

## Conclusion

The objective of The XRCISE-AS-INSTRUcted-1 trial is to determine if live feedback on TUT will improve the ability to perform the exercises as prescribed among adolescents with PFP. This could potentially solve the problem of low compliance in this patient group.

## Abbreviations

DOMS, Delayed Onset Muscle Soreness; GP, General Practice; ICC, Intraclass Correlation Coefficient; ITT, Intention-To-Treat; PFP, Patellofemoral Pain; ROM, Range Of Motion; RR, Relative risk; RM, Repetition Maximum; TUT, Time Under Tension; VAS, Visual Analog Scale
